# Assessing 5-year follow-up of core outcome set uptake for Bronchiectasis and Hidradenitis Suppurativa: a review of trial registry entries

**DOI:** 10.1136/bmjopen-2024-095190

**Published:** 2025-08-18

**Authors:** Charlotte Shorey, Paula R Williamson, Susanna Dodd

**Affiliations:** 1 University of Liverpool, Liverpool, UK; 2 Health Data Science, University of Liverpool, Liverpool, UK

**Keywords:** DERMATOLOGY, RESPIRATORY MEDICINE (see Thoracic Medicine), Research Design, STATISTICS & RESEARCH METHODS

## Abstract

**ABSTRACT:**

**Objective:**

If clinical trials measure and report the outcomes included in core outcome sets (COS) for a given condition/disease as a minimum, this has the potential to improve comparability between trials and prevent research waste. Until now, the uptake of the Bronchiectasis and Hidradenitis Suppurativa (HS) COS has not been assessed.

This study assessed the uptake of Bronchiectasis and HS COS using a review of trial registries, with entries taken from ClinicalTrials.gov and the WHO International Clinical Trial Registry Platform. This uptake assessment provides valuable information to inform COS refinement and uncover areas lacking uptake to inform further dissemination requirements.

**Methods:**

For each trial, the outcomes included in the trial registry entry were extracted and compared with those included in the corresponding Bronchiectasis or HS COS. The Bronchiectasis COS consists of 18 outcomes, and the HS COS, 6.

**Results:**

Of the trials registered after both COS were developed in 2018, 63% (12/19) of HS trials planned to measure the full COS, whereas for Bronchiectasis, 0% (0/24) of trials planned to measure the full COS. However, of the five priority outcomes to be measured for Bronchiectasis, 4% (1/24) of trials planned to measure all five outcomes.

Both COS publications’ focus was to reach consensus on what outcomes should be measured. Despite both publications referring to the Core outcome Measures for Effectiveness Trials (COMET) Handbook, which discusses the importance of COS dissemination, implementation plans were not included in either publication.

**Conclusions:**

The results suggest that uptake of the HS COS is relatively good, despite yearly fluctuations, whereas for Bronchiectasis, COS uptake is limited. Further research into standardised measurement tools for HS is expected to increase uptake. The focus for Bronchiectasis, however, will be to refine the COS for feasible application in clinical trials. Future COS development publications should use all resources from the COMET initiative to ensure feasible dissemination of the COS.

**STRENGTHS AND LIMITATIONS OF THIS STUDY**

- This study assessed core outcome set (COS) uptake in all trials relevant to each COS over a 5-year period both before and after COS publication.
- Outcome matching between the COS and the registry entry was checked by an experienced methodologist in this field.
- Trialists were not contacted to assess their explicit awareness of COS.

## Introduction

 For healthcare to benefit from the research efforts of clinical trials, it is crucial that appropriate outcomes are chosen to be measured and reported. This is important to ensure that they are relevant to patients, public, healthcare professionals, and those involved in healthcare decision-making. This issue can be addressed by utilisation of a core outcome set (COS), which is defined as an agreed standardised set of outcomes that should be measured and reported as a minimum, in all clinical trials in a specific area of health or healthcare.^1^ To ensure relevance, key stakeholders like patients, clinicians and researchers should be involved in COS development.

The Core outcome Measures for Effectiveness Trials (COMET) initiative^2^ was created to raise awareness of why COS is necessary, encouraging standardised, evidence-based COS development and uptake. This initiative has many resources available for COS developers to use, such as the COMET handbook,^3^ Core Outcome Set STAndards for Development, Core Outcome Set STAndardized Protocol Items and Core Outcome Set STAndards for Reporting. Well-designed COS use these resources to ensure they are developed effectively.

As COS are developed to be disease-specific, the chosen outcomes are relevant, ensuring all trials contribute useful information. Moreover, as studies are more likely to report statistically significant results to increase their chances of being published,^4^ uptake of COS by trialists will prevent such outcome reporting biases, as it would become standard practice to report all outcomes in the COS, as a minimum. Furthermore, COS limit the risk of research waste for clinical trials by providing standardisation of measured and reported outcomes, thereby facilitating comparability and combining of results across trials.

Yet, for trials to benefit from the standardisation of measured and reported outcomes, COS uptake must occur, as if COS are not utilised by trials, COS themselves lead to research waste, contradictory to their purpose. Uptake assessments for COS are important to identify whether they are preventing or adding to research waste, and if the latter, to prompt action in terms of wider dissemination and implementation of COS.

Currently, there are few studies assessing COS uptake in clinical trials^5 6^; thus more research is needed to assess whether the implementation of COS is occurring across all disease categories.

In 2018, COS for two diseases, Bronchiectasis^7^ and Hidradenitis Suppurativa (HS)^8^, were developed. This study aimed to assess the extent of COS uptake in clinical trials for these two COS using a review of trial registry entries. These COS were selected for uptake assessment, as they were both well developed (with patient participation) and had a sufficiently long enough period of follow-up since publication (5 years, at the time this study commenced) to allow comprehensive uptake assessment. Previous COS uptake studies have involved relatively common diseases, such as rheumatoid arthritis,^5^ whereas this study sought to demonstrate methods and uptake for less prevalent diseases. Both disease COS are shown in table 1.

**Table 1:** Finalised core outcome sets (COS) for Bronchiectasis and Hidradenitis Suppurativa (HS).

Bronchiectasis^7^	HS^8^
Serious adverse effects	Pain
Death (disease)	Physical signs
Pulmonary exacerbations	HS-specific quality of life
Admissions to hospital	Global assessment
Quality of life	Progression of course
Death (all-cause)	Symptoms
Adverse effect: shortness of breath	
Adherence to treatment	
Sputum characteristics	
Sputum microbiology	
Lung function	
Shortness of breath	
Haemoptysis	
Cough	
Exercise tolerance	
Patient perception of health	
Accident and emergency (A & E) attendances	
Activities of daily living	

For the HS COS, an extra outcome of ‘symptoms’ reached ‘consensus-in’ from patients but not healthcare professionals. However, this outcome was nevertheless considered to be important in the COS, as the steering committee decided that the patient voice superseded the healthcare professional vote when considering patient-reported outcomes, and was therefore recommended to be measured on a case-by-case basis. This outcome has also, therefore, been included for uptake assessment in this study, due to the importance of the patient voice and patient-reported outcomes in research.^9^ For Bronchiectasis, the study recommended that the top five outcomes should be prioritised for uptake, with the remaining outcomes considered on a case-by-case basis. Both the top five outcomes and the full set of 18 outcomes were assessed here for uptake.

## Methods

### Trial extraction

Clinical trials should be registered with trial registries such as ClinicalTrials.gov, thereby being gathered onto larger, international registry platforms like the WHO International Clinical Trials Registry Platform (WHO ICTRP). Registering trials prevents unnecessary duplication of research efforts and selective reporting of research outcomes as they must be prespecified. Both of these sources were used to identify relevant trials and their outcomes for HS and Bronchiectasis, to provide an efficient and accurate estimate for assessing uptake.^5 10^

Phase 3 and 4 trials for each condition were searched for using registry posting dates, which allowed for sufficient trial entries to be retrieved (see online supplemental file 1 for more details).

The original aim was to assess trials by comparing a similar timeframe (namely 5 years) before and after COS development; however, for both diseases, especially HS, few pre-COS trials (according to date of trial registration) began within this time period. To overcome this, trials were retrieved from an earlier date to ensure sufficient numbers of pre-COS trials to allow meaningful comparison.

### Inclusion and exclusion of trials

Once retrieved, the trial registry entries were exported to a spreadsheet and filtered via the inclusion and exclusion criteria as stated in each COS. Trials were manually excluded if the disease mentioned was not HS or Bronchiectasis. The condition information was also scrutinised to ensure they met the criteria of the disease-specific COS. The Bronchiectasis COS specified its suitability to trials involving adults only and so trials involving participants <18 years were excluded. After excluding non-applicable trials, duplicate trial entries (registered in both the WHO ICTRP and ClinicalTrials.gov) were manually filtered by comparison of trial IDs. The remaining trials from each source were then combined. This was completed separately for both diseases.

### Outcome matching and assessment of COS uptake

As with previous COS uptake assessments,^5 10 11^ results are presented descriptively, with no statistical analyses to synthesise the data.

After all trial outcomes were extracted from trial registry entries and assessed for matching to the COS, each trial was scored out of 6 for HS and 18 (and five) for Bronchiectasis (full and recommended COS, respectively) to represent the number of matches to the disease-specific COS.

When deciding whether an outcome matches the COS or not, the outcome measured by the trial must be fully understood. For example, trials may state a composite outcome/measurement tool such as a questionnaire assessing quality of life, as opposed to mentioning the COS outcome ‘Quality of Life’. In this instance, the measurement tool was scrutinised to determine which outcomes were included; for example the Leister Cough Questionnaire for Bronchiectasis received a match for ‘Quality of Life’, ‘Cough’, ‘Sputum’, ‘Activities of Daily Living’, and ‘Patient Perception of Health’ as the questionnaire additionally addresses these domains.

Additionally, for Bronchiectasis, the ‘Shortness of Breath’ and ‘Adverse effect: Shortness of breath’ outcomes have been considered equivalent—as it is not possible to distinguish between these from trial reports.

### Patient and public involvement

Patients and public were not involved in the design of this uptake assessment. However, patients participated in the development of both of the COS that were assessed in this study and this is considered an important standard of quality in COS development, according to COS-STAD recommendations.^12^

## Results

### Overview

It is expected that trials registered after their respective COS were published will be more likely to measure the COS outcomes. Of the trials registered after COS publication, 63% (12/19) of HS trials were planned to measure the full COS, whereas for Bronchiectasis, 0% (0/24) of trials were planned to measure the full COS of 18 outcomes. Of the five priority outcomes to be measured for Bronchiectasis, 4% (1/24) of trials were planned to measure all five outcomes.

### Extracted trials

#### Hidradenitis Suppurativa

**Figure 1:**
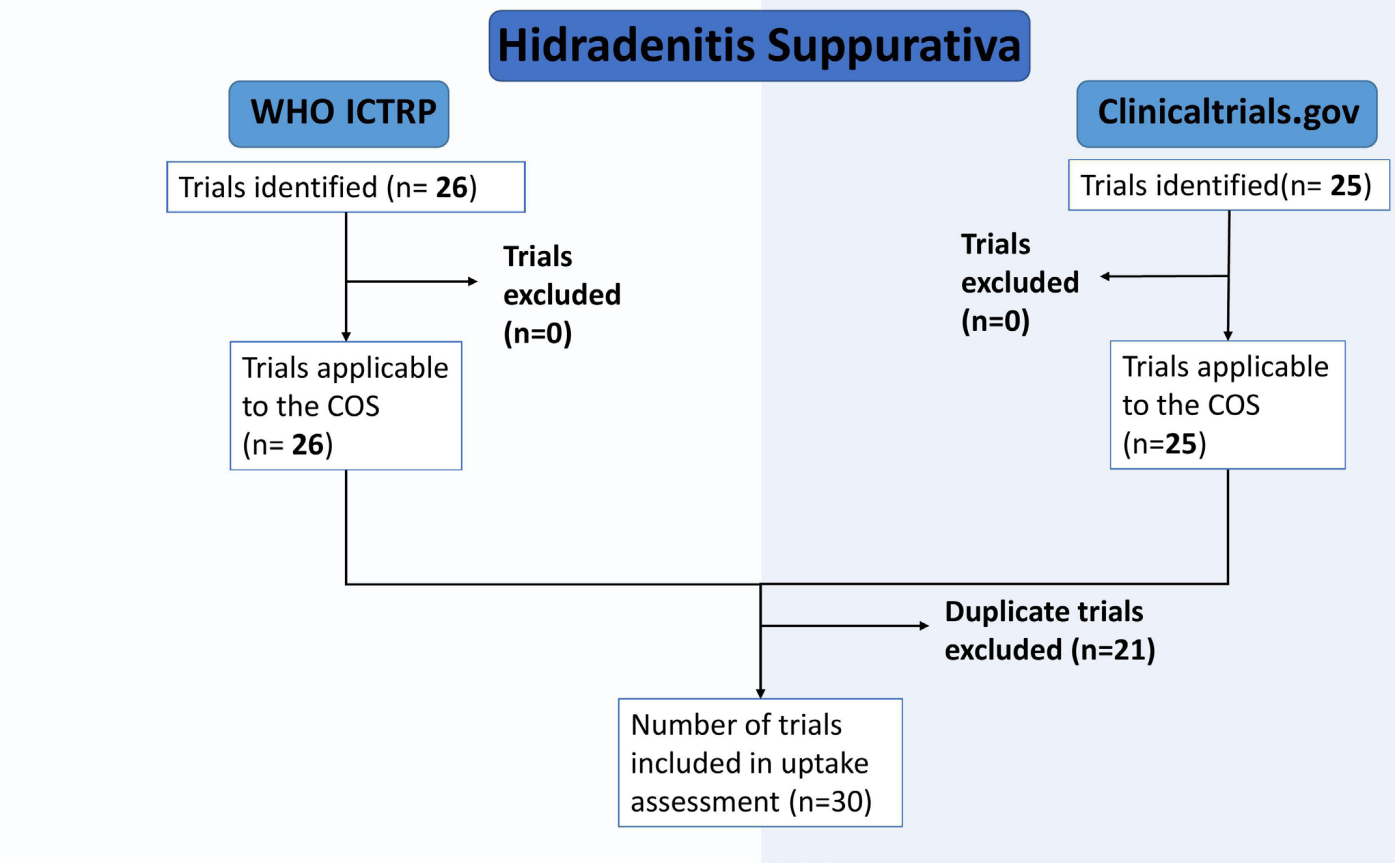
Flow diagram of the selection process for eligible Hidradenitis Suppurativa (HS) trials. COS, core outcome sets; WHO ICTRP, The WHO Clinical Trials Registry Platform.

Using the search strategy shown in online supplemental file 1, 51 trials were identified. After filtering trials for relevance to the COS, no trials were ineligible; however, there were 21 duplicates between the trials retrieved from ClinicalTrials.gov and WHO ICTRP searches, leaving 30 unique trials overall. This filtering process is as seen in figure 1.

Of the 30 trials included in the uptake assessment, 21 trials appeared in both searches as duplicates, five were unique to the WHO ICTRP, and four were unique to ClinicalTrials.gov.

#### Bronchiectasis

**Figure 2:**
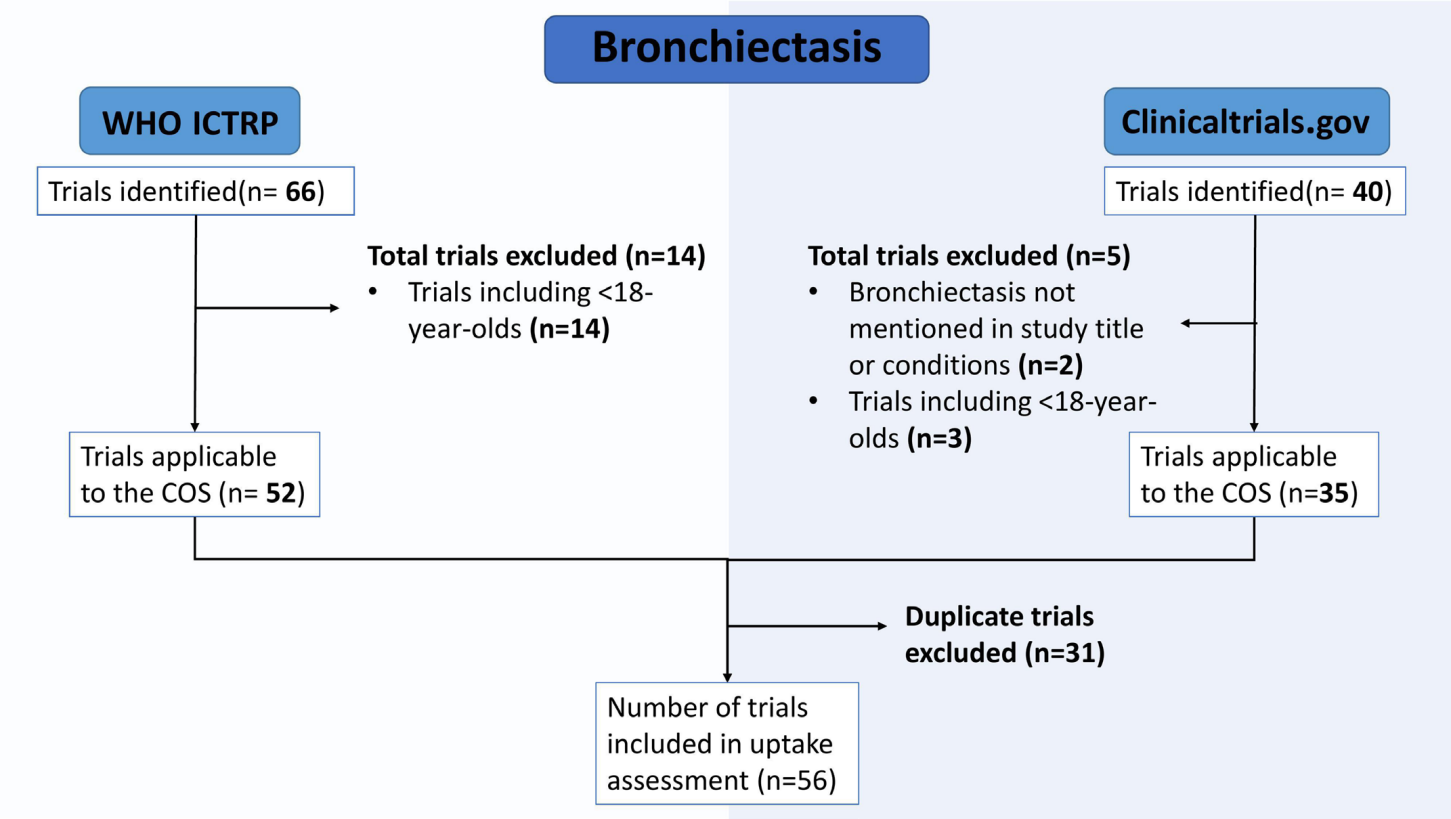
Flow diagram of the selection process for eligible Bronchiectasis trials. COS, core outcome sets; WHO ICTRP, The WHO Clinical Trials Registry Platform.

Using the parameters included in online supplemental file 1, 106 trials were identified. After filtering trials for relevance to the COS, 19 trials were excluded: 17 included under 18-year olds, and 2 did not mention the disease in the study title or conditions. Between the trials retrieved from ClinicalTrials.gov and the WHO ICTRP, there were 31 duplicates, leaving 56 trials overall. This filtering process is shown in figure 2.

Of the 56 trials, 31 trials appeared in both searches as duplicates, 21 trials were unique to the WHO ICTRP, and 5 were unique to ClinicalTrials.gov.

### Reporting of the disease-specific COS

#### Hidradenitis Suppurativa

**Figure 3:**
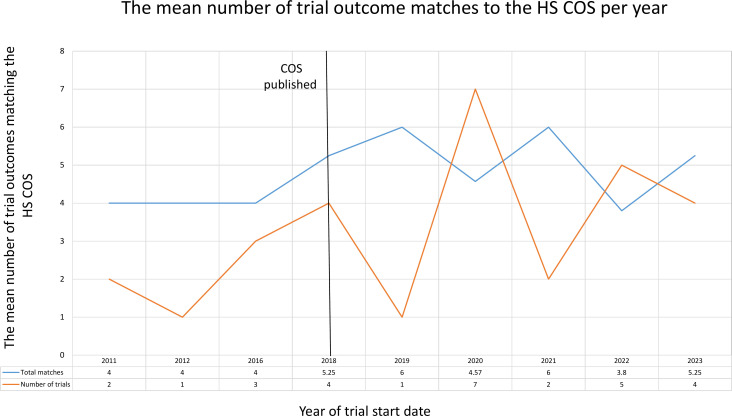
The mean number of trial outcomes matching the full Hidradenitis Suppurativa Core Outcome Set (HS COS) from 2005 to 2023.

The uptake of the HS COS is illustrated in figure 3. This shows the yearly mean number of HS COS outcomes, which were planned to be measured by trials before and after publication of the COS in 2018.

As there was only one trial identified in 2005, and no more trials up until 2011, the 2005 trial has not been included in the graph.

An upward trend between 2016 and 2019 is present, followed by fluctuations up until 2023. Of the 19 trials registered after 2018, 63% (12/19) of trials were planned to measure the full COS; 5% (1/19) were planned to measure four outcomes, 16% (3/19) three outcomes, 11% (2/19) two outcomes, and 5% (1/19) one outcome.

Despite the outcome ‘symptoms’ only being recommended to be measured on a case-by-case basis, the outcome has still been measured by 63% (12/19) of trials registered after COS publication. This suggests trialists are recognising the importance of patient-reported outcomes in research.^8^

#### Bronchiectasis

**Figure 4:**
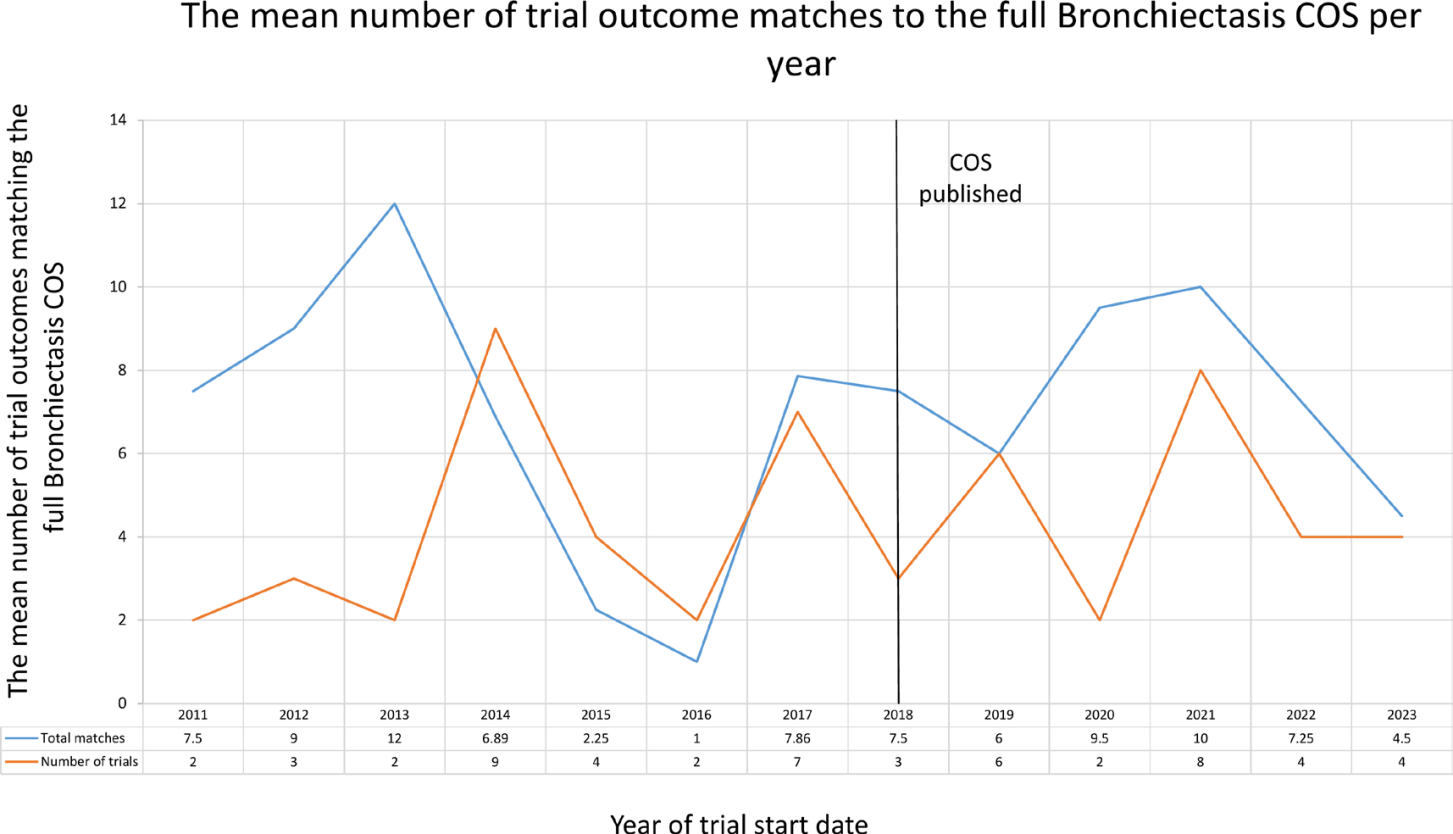
The mean number of trial outcomes matching the full Bronchiectasis core outcome set (COS) from 2011 to 2023.

The uptake of the Bronchiectasis COS is illustrated in figure 4. This shows the yearly mean number of Bronchiectasis COS outcomes which were planned to be measured by trials before and after publication of the COS in 2018.

No clear upward trend can be seen here with great fluctuations over the uptake timeline. No trial was planned to measure all 18 outcomes. Of the 24 trials registered after COS publication, there is a range of 0–15 outcomes being included in trial registry entries, with a mode of 10 (4 trials were planned to measure 10 outcomes).

**Figure 5:**
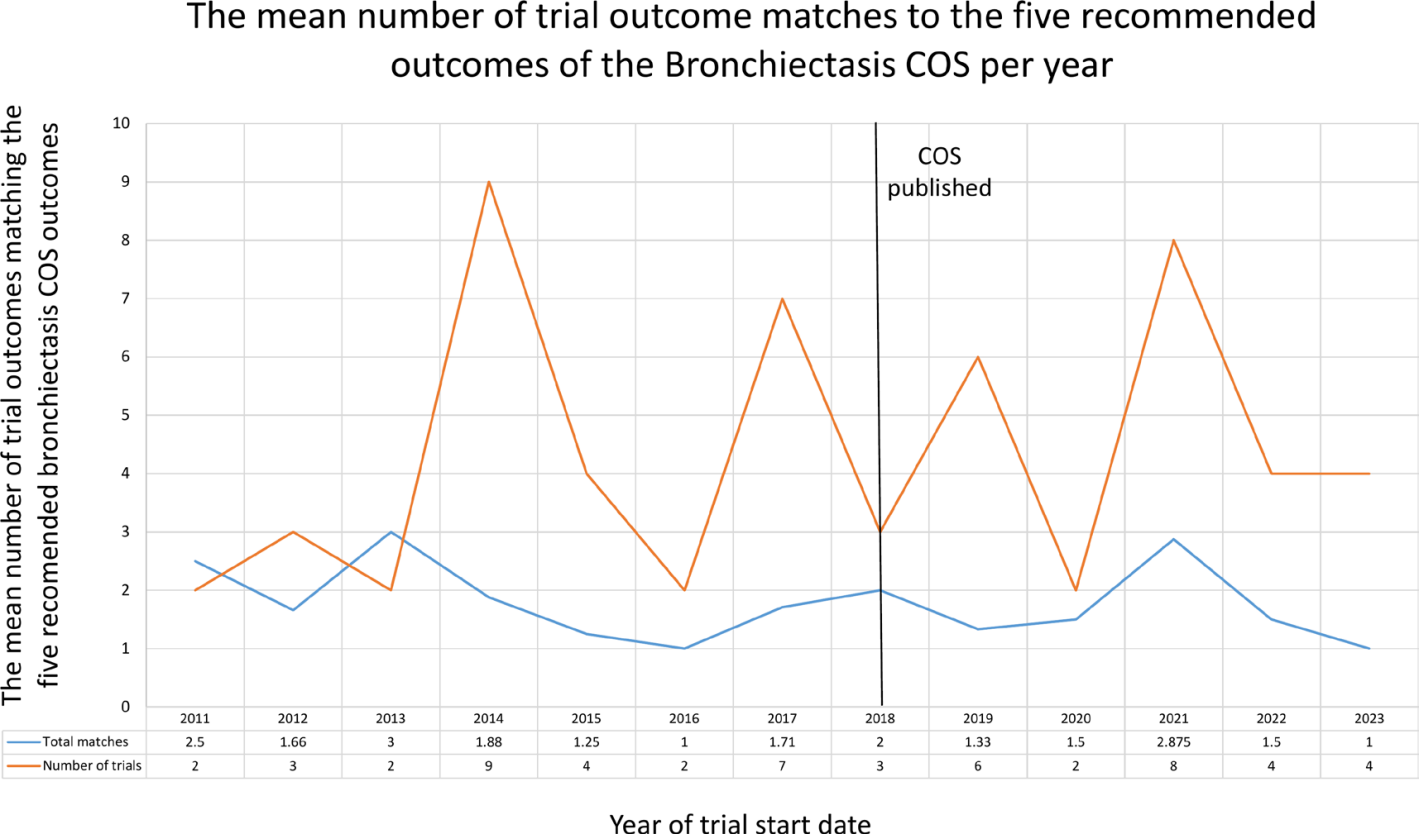
The average number of trial outcomes matching the five recommended Bronchiectasis core outcome set (COS) from 2011 to 2023.

The uptake of the top five Bronchiectasis COS outcomes is illustrated in figure 5.

Of the 24 trials registered after COS publication, only 4% (1/24) of trials were planned to measure the full five recommended outcomes, 8% (2/24) four outcomes, 12% (3/24) three outcomes, 29% (7/24) two outcomes, 33% (8/24) one outcome and 12% (3/24) no outcomes.

There is no substantial evidence of increased uptake since the publication of the COS in 2018 as the yearly mean uptake shown in figure 5 does not exceed three of the five recommended outcomes.

## Discussion

This study has identified that although there are fluctuations in the yearly mean uptake of the HS COS, there are trials which were planned to measure the full COS. However, for the Bronchiectasis COS, there is limited uptake as no trials were planned to measure the full COS and only one trial was planned to measure all five priority outcomes. As there are a limited number of trials, small fluctuations in results may appear as impactful findings; however, strong conclusions cannot be deduced from minor changes in the outcome matching means per year. Moreover, for COS to have an impact in a limited number of trials, its uptake must be accelerated.

This uptake assessment could indicate relatively high awareness of the HS COS as more trials measure a larger proportion of the COS. However, to gauge awareness, it would be helpful to review the subsequent trial publications to identify if they explicitly cite the COS. Furthermore, reviewing trial publications would also help to determine whether selective reporting has occurred, by assessing whether there are discrepancies between outcomes listed in the trial registry entry compared with those reported in the publication. Additionally, as Kirkham *et al*^12^ suggest, trialists who failed to list any outcomes are likely to have simply failed to fully record their trial information in the registries; therefore, targeted contact with these individual trialists would help to determine their awareness of the COS.

For Bronchiectasis, there is limited indication of sufficient COS uptake as the outcome matching trends over the years fluctuate, thus not illustrating consistent uptake improvements. This applied to both the full COS and the five priority outcomes recommended by Spargo *et al*.^7^ Like with HS, to gauge explicit awareness, the subsequent trial publications could be assessed to identify whether they cite the COS, and trialists could be contacted where zero outcomes were included in the trial registry entries.

Reasons for limited uptake have previously been discussed,^5^ including the number of outcomes, lack of clarity, lack of validated measures and lack of COS awareness. Although there is no recommended minimum or maximum number of outcomes, it can be assumed this should be limited to ensure feasible COS uptake in clinical trials. As the HS COS has six outcomes, immediate implementation is not an issue, whereas for Bronchiectasis, the 18-item list must be refined for application in clinical trials. Spargo *et al*^7^ thus recommended uptake of the top five outcomes, yet those outcomes also lacked consistent uptake. Moreover, on searching the COMET database and PubMed, no new studies addressing refinement of this Bronchiectasis COS were identified.

Additionally, for Bronchiectasis, one feature of the COS which remained ambiguous was the inclusion of both ‘shortness of breath’ and ‘adverse effect: shortness of breath’. Dodd *et al*^13^ stated that adverse events listed specifically are not considered to fall within the adverse event domain, thus making the latter outcome redundant. Removal of this outcome would, therefore, help to reduce confusion and further refine the COS list towards feasible uptake.

Lack of uptake relating to specific outcomes may not result from lack of relevance to the disease but instead as a result of other barriers such as confusion regarding how to measure each outcome, caused by limited standardisation of measurement tools for both diseases.^14^,^16^

Research is currently being carried out by Hidradenitis Suppurativa Core Outcomes Set International Collaboration^17^ (HiSTORIC) to ensure relevant, standardised measurement tools for each of the COS outcomes are identified (HiSTORIC, The CHORD COUSIN Collaboration). This initiative is similar to Outcome Measures in Rheumatoid Arthritis Clinical Trials (OMERACT), which could also explain the higher uptake of HS COS compared with Bronchiectasis, which lacks such an initiative. Before measurement tools for Bronchiectasis can be standardised, focus should be directed towards refining the COS, and a similar supporting group to OMERACT should be developed to ensure future research is monitored and the outreach of the COS is increased.

In reviewing trial registry records rather than trial protocols or publications, this study sought to determine planned rather than actual outcome measurement. However, the advantage of using registry records to determine COS uptake is that researchers are thereby assessing current trends, whereas publications and protocols tend to reflect outcome decisions that were made many years previously.

This study also highlights the importance of utilising more than one trial source, as neither of the two sources used to collect trial results identified the full cohort of trials. The WHO-ICTRP collates information from trial registries across the globe, allowing the search portal to identify many, geographically diverse clinical trials, thereby facilitating comprehensive uptake assessment.^18^ Despite ClinicalTrials.gov being an American trial registry, it also attracts registration from studies conducted around the world, providing another platform from which to assess COS uptake globally.^12^ However, although ClinicalTrials.gov and the WHO-ICTRP individually provide a reasonable estimate of uptake, both sources failed to identify the full cohort of trials which had been registered. Therefore, in order to identify all relevant trials, especially for relatively uncommon diseases with limited number of trials like HS, the use of both sources is recommended to ensure accurate assessment.

## Conclusion

This is the first study assessing the uptake of the HS and Bronchiectasis COS. Neither disease exhibits consistent uptake of the full COS; however, uptake of the HS COS is promising as the full COS is being measured by some trials. Further research into standardised measurement tools for HS is expected to increase uptake. The focus for Bronchiectasis, however, will be to refine the COS for feasible application in clinical trials. This study also emphasises the importance of using multiple trial registry platforms to identify all relevant trials, to comprehensively assess COS uptake, especially for diseases with limited trials.

## Supplementary material

10.1136/bmjopen-2024-095190 online supplemental file 1

## Data Availability

Data are available upon reasonable request.
