# Supplementary material for: Assessing 5-year follow-up of core outcome set uptake for Bronchiectasis and Hidradenitis Suppurativa: a review of trial registry entries
Source: BMJ Open. 2025 Aug 18;15(8):e095190. doi: 10.1136/bmjopen-2024-095190 (PMC12366604; doi:10.1136/bmjopen-2024-095190)
Supplement: online supplemental file 1 [file bmjopen-15-8-s001.docx]

**Supplementary material: Search strategy**

The HS COS was developed on July 5^th^ 2018 [8] and the bronchiectasis COS on 3^rd^ October 2018 [7].

| **Search Type** | **Input** |
| --- | --- |
| Recruitment status | All |
| Study type | Interventional studies |
| Disease/condition | Bronchiectasis/Hidradenitis Suppurativa (respectively) |
| Phase | 3 and 4 |
| Dates | First posted from  **Bronchiectasis:** 1^st^ January 2012 to 14^th^ July 2023 (date of search)  **Hidradenitis Suppurativa:** 1^st^ January 2006 to 14^th^ July 2023 (date of search) |
