# Supplementary material for: Assessing 5-year follow-up of core outcome set uptake for Bronchiectasis and Hidradenitis Suppurativa: a review of trial registry entries
Source: BMJ Open. 2025 Aug 18;15(8):e095190. doi: 10.1136/bmjopen-2024-095190 (PMC12366604; doi:10.1136/bmjopen-2024-095190)
Supplement: Supplementary file 2 [file bmjopen-2024-095190.reviewer_comments.pdf]

PEER REVIEW HISTORY

BMJ Open publishes all reviews undertaken for accepted manuscripts. Reviewers are asked to complete a checklist review form and are provided with free text boxes to elaborate on their assessment. These free text comments are reproduced below.

ARTICLE DETAILS

Title (Provisional)

Assessing 5-year follow up of Core Outcome Set uptake for Bronchiectasis and Hidradenitis Suppurativa: a review of trial registry entries

Authors

Shorey, Charlotte; Williamson, Paula R; Dodd, Susanna

VERSION 1 - REVIEW

**Reviewer**	**1**
**Name**	**Shipman, William D**
**Affiliation**	**Yale University School of Medicine**
**Date**	**08-Jan-2025**
**COI**	

The authors present a manuscript analyzing the core outcome sets of bronchiectasis and HS but provide no clear basis for why this analysis is important and meaningful. Unfortunately this seems to be simply a precursory search of the Clinical Trial databases but really has no clinical importance or relevance. I do not understand how bronchiectasis and HS are related or why these two conditions were chosen.

**Reviewer**	**2**
**Name**	**Ingram, John**
**Affiliation**	**Cardiff University**
**Date**	**01-Jun-2025**

COI **Dr Ingram received a stipend as immediate past-Editor-in-Chief of the British Journal of Dermatology and an authorship honorarium from UpToDate. He is a consultant for Abbvie, Boehringer Ingelheim, Cantargia, ChemoCentryx, Cityrll, Elasmogen, Engitix, Incyte, Indero, Insmed, Kymera Therapeutics, MoonLake, Novartis, UCB Pharma, UNION Therapeutics, and Viela Bio. He is co-copyright holder of HiSQOL, Investigator Global Assessment and Patient Global Assessment instruments for HS and his**

department receives income from copyright of the Dermatology Life Quality Index (DLQI) and related instruments. He is Treasurer of the CHORD-COUSIN Collaboration (C3) dermatology outcomes consortium and a co-founder of HISTORIC.

The manuscript is a helpful comparison on the uptake of two COS, in the context that implementation is a key element of COS impact. The manuscript is well written and adds to the literature. I have only minor comments:

When reviewing the title, I wasn't sure why HS and bronchiectasis were the two COS selected because they don't seem to be related conditions. On reading the article the reasons for picking the two conditions become clearly apparent, in particular publication of both COS in 2018. Perhaps this could be made clearer in the title by including something like '10-year follow up of COS for...' or 'A comparison of two COS developed in 2018 for HS and bronchiectasis...'

Regarding the HS symptoms domain, this is included in the HS COS because the Steering Committee decided that the patient voice superseded the HCP vote for a PROM.

To help with implementation of the HS COS, perhaps the website address could be included in the article: https://www.c3outcomes.org/historic

---

VERSION 1 - AUTHOR RESPONSE

Reviewer 1's comments:

- The authors present a manuscript analyzing the core outcome sets of bronchiectasis and HS but provide no clear basis for why this analysis is important and meaningful. Unfortunately this seems to be simply a precursory search of the Clinical Trial databases but really has no clinical importance or relevance. I do not understand how bronchiectasis and HS are related or why these two conditions were chosen. Thank you for this comment. The rationale for COS choice has been clarified in the introduction, namely that these COS were selected for uptake assessment as they were both well developed (with patient participation) and had a sufficiently long enough period of follow up since publication (five years, at the time this study commenced) to allow comprehensive uptake assessment. Previous COS uptake studies have involved relatively common diseases, such as rheumatoid arthritis, whereas this study sought to demonstrate methods and uptake for less prevalent diseases.

Reviewer 2's comments:

- The manuscript is a helpful comparison on the uptake of two COS, in the context that implementation is a key element of COS impact. The manuscript is well written and adds to the literature. I have only minor comments: Thank you for these comments.

- When reviewing the title, I wasn't sure why HS and bronchiectasis were the two COS selected because they don't seem to be related conditions. On reading the article the

reasons for picking the two conditions become clearly apparent, in particular publication of both COS in 2018. Perhaps this could be made clearer in the title by including something like '10-year follow up of COS for...' or 'A comparison of two COS developed in 2018 for HS and bronchiectasis...' Thank you for this suggestion. The addition of "5-year follow up of COS uptake" has been added to the title, as this was the time period post-COS publication available for uptake assessment at the time of the original trial registry search.

- Regarding the HS symptoms domain, this is included in the HS COS because the Steering Committee decided that the patient voice superseded the HCP vote for a PROM. Thank you for this information, which has been added to the introduction section.

- To help with implementation of the HS COS, perhaps the website address could be included in the article: https://www.c3outcomes.org/historic Thank you for this useful reference – it has been added in the discussion of the work being conducted by the HiSTORIC initiative.
